# Phytic Acid-Containing Reactive Acrylic Emulsions in Leather Coating Applications

**DOI:** 10.3390/polym17212905

**Published:** 2025-10-30

**Authors:** Kaan Canli, Catalina N. Cheaburu-Yilmaz, Raluca Nicoleta Darie-Nita, Onur Yilmaz

**Affiliations:** 1 Leather Engineering Department, Faculty of Engineering, Ege University, 35100 Izmir, Türkiye; kaan.cnll00@gmail.com; 2 Chemistry Department, Tınaztepe Campus, Dokuz Eylul University, 35390 Izmir, Türkiye; catalinanatalia.yilmaz@deu.edu.tr; 3 Physical Chemistry of Polymers Department, “Petru Poni” Institute of Macromolecular Chemistry, 41A Gr. Ghica Voda Alley, 700487 Iasi, Romania

**Keywords:** reactive acrylic polymer, phytic acid, leather finishing, binder, emulsion polymerization, glycidyl methacrylate, vinyltriethoxysilane

## Abstract

Phytic acid, as a natural originated compound with multi phosphate side groups, is known to increase the corrosion protection and thermal resistance of the coatings. In this study, two different acrylic emulsion polymers containing epoxy and silane reactive functional groups (glycidyl methacrylate (GMA) and vinyltriethoxysilane (VTES)) were synthesized via emulsion polymerization and mixed with phytic acid (PA) solution in different ratios (5, 10, 15 wt%) for use as binders in leather finishing applications. The colloidal stability, particle size distribution, and chemical structures of the synthesized polymers were characterized through comprehensive analyses. The resulting reactive copolymer dispersions were used as binders in finishing formulations and applied to crust shoe upper leathers The coating performance was evaluated in terms of rub fastness, flex resistance, water spotting, and thermal resistance, using the unmodified reactive acrylic binders (G0 and V0) as reference systems to assess the improvements achieved. Both phytic acid-modified binders exhibited strong film integrity and maintained high dry rub fastness up to 2000 cycles and wet rub fastness up to 250 cycles at phytic acid concentrations of 5–10 wt%. Increasing the phytic acid content beyond this range led to reduced dispersion stability and partial loss of coating performance. The results confirm that incorporating moderate levels of phytic acid into reactive acrylic emulsions enhances coating durability and thermal resistance without compromising film appearance, offering a safer and more sustainable alternative to conventional crosslinking systems for leather finishing applications.

## 1. Introduction

Leather finishing is the final and a critical stage of leather processing, providing the surface with desired appearance, handle, and functional performance. To enhance the mechanical strength, chemical resistance, and durability of finishing films, crosslinking agents are commonly incorporated into polymeric binders. Crosslinking generates covalent bridges between polymer chains, resulting in a three-dimensional network that improves abrasion and heat resistance while reducing film solubility [1,2,3]. Conventional crosslinkers such as formaldehyde, isocyanate-based agents, and aziridine derivatives have been widely used for this purpose. However, these compounds are associated with toxicity, allergenicity, and volatile organic compound (VOC) emissions, raising serious concerns regarding worker safety, environmental sustainability, and compliance with increasingly strict regulatory frameworks [4]. Consequently, the development of non-toxic, eco-friendly crosslinking systems has become a key focus in the leather and textile finishing industries.

One promising strategy involves the use of reactive monomers that enable self-crosslinking or post-crosslinking within the polymer backbone. Epoxy- and silane-functional monomers are particularly attractive in this regard [4,5]. Glycidyl methacrylate (GMA) contains epoxy groups that readily undergo nucleophilic ring-opening reactions under acidic or basic conditions, facilitating covalent crosslink formation [6,7,8]. Similarly, vinyltriethoxysilane (VTES) introduces alkoxysilane moieties that hydrolyze to silanols and subsequently condense to produce siloxane (Si–O–Si) linkages, yielding robust organic–inorganic hybrid networks [9,10,11]. Incorporating such reactive functionalities into acrylic copolymers has been shown to improve adhesion to the leather substrate and to enhance properties such as wet rub fastness, flexibility, and chemical resistance [12,13].

Phytic acid (myo-inositol hexakisphosphate, PA) is a naturally occurring organophosphorus compound abundant in plant seeds and grains. Biologically renewable and non-toxic, PA contains six phosphate groups capable of strong chelation with multivalent metal ions, endowing it with multifunctional characteristics including antioxidant, antimicrobial, corrosion-inhibiting, and flame-retardant effects [14,15]. In particular, PA exhibits anticorrosion properties by chelating metal ions to form protective phosphate layers on surfaces, while its high phosphorus content facilitates the formation of thermally stable char, providing flame retardancy and enhanced thermal stability.

Recent studies have further demonstrated the versatility of PA in coating systems. Hybrid sol–gel formulations combining PA with silica precursors improved the thermal stability and flame retardancy of cotton fabrics by forming phosphorus–silicon synergistic networks [15,16,17,18]. PA–silanol hybrids have also been used to protect magnesium alloys and steel substrates, where strong metal–phosphate chelation contributed to enhanced corrosion resistance and even self-healing behavior [19,20]. Moreover, PA-modified graphene and conductive polymers have been incorporated into waterborne polyurethane or epoxy matrices to achieve superior barrier properties and active corrosion inhibition [21,22,23,24]. More specifically, in waterborne polymer coatings, PA has been shown to increase crosslinking density and film cohesion, thereby improving hardness, water resistance, and adhesion strength. Liu et al. [25] reported that PA-modified waterborne coatings exhibited markedly enhanced corrosion protection, while Aljamal [26] demonstrated that PA served as both curing agent and flame retardant in a water-based epoxy system. In another study, Böhm et al. [27] highlighted strategies such as hydrophobization and oligomerization to improve PA compatibility with conventional coating resins, presenting PA as a sustainable crosslinking candidate for advanced polymer networks, especially for epoxy and polyol systems. These findings collectively underline PA’s unique ability to act as a bio-based, multifunctional performance enhancer in waterborne coating systems.

Building on these developments, the present work focuses on the synthesis and application of phytic-acid-modified reactive acrylic binders specifically designed for leather finishing—a field in which phytic acid is introduced for the first time as a reactive, bio-based crosslinking component. While phytic acid has been explored in textile and coating formulations, its incorporation into epoxy-functional (BA–MMA–GMA) and alkoxysilane-functional (BA–MMA–VTES) acrylic latexes represents a novel, environmentally benign strategy to replace conventional toxic crosslinkers. These two reactive systems provide crosslinking and hydrogen bonding mechanisms—epoxy–phosphate interactions in GMA-based copolymers and silanol–phosphate coordination in VTES-based copolymers—enhancing coating cohesion, film integrity, and adhesion to chromium-tanned leather substrate.

The study systematically investigates the effect of phytic acid incorporation on colloidal stability, interfacial film formation, and mechanical performance of the binders. Particular attention is given to rub fastness, flex resistance, water spotting, water and solvent uptake, and thermal resistance, providing a comprehensive evaluation of coating durability. Overall, the developed phytic-acid-reactive acrylic systems offer a sustainable, high-performance alternative to conventional crosslinking approaches in advanced leather finishing.

## 2. Materials and Methods

### 2.1. Materials

For the synthesis of the reactive acrylic emulsion polymers; methyl methacrylate (MMA, ≥98.5%), butyl acrylate (BA, ≥99.0%), glycidyl methacrylate (GMA, ≥97%) and vinyltriethoxysilane (VTES, ≥98%) were purchased from Sigma-Aldrich (Merck KGaA, Darmstadt, Germany) and used as monomers. To stabilize the emulsion system, a combination of anionic and nonionic surfactants—Sulfopon^®^ 101 UP (28–30%, BASF, Ludwigshafen, Germany) and Emulsogen EPN 287 (68–70%, Clariant, Burgkirchen, Germany)—was employed. Ammonium persulfate (APS, 99.0%, Sigma-Aldrich, Darmstadt, Germany) was used as the initiator and sodium bicarbonate (NaHCO_3_, 99.0%, Merck, Darmstadt, Germany) as a buffering agent to maintain pH stability. All polymerizations were conducted in a four-neck glass reactor equipped with a reflux condenser, dropping funnels and mechanical stirring. The reaction temperature was controlled with an IKA (Staufen, Germany) thermostated heater. A glycerin bath was used to maintain constant reaction temperature during polymerization.

Phytic acid (PA, 50% aqueous solution, Sigma-Aldrich, Darmstadt, Germany) was used as a bio-based binder additive. The PA solution was partially neutralized to pH = 3.5–4.0 by 10 M NaOH solution before use.

For leather finishing applications, chromium tanned crust leather specimens (bovine, shoe-grade) were supplied by Sepiciler A.Ş. (İzmir, Türkiye). Auxiliary finishing chemicals—including wax emulsion, brown pigment, and nitrocellulose-based topcoat—were obtained from Stahl B.V. (Waalwijk, The Netherlands) and applied in combination with the synthesized reactive copolymer dispersions.

### 2.2. Synthesis of Reactive Acrylic Emulsion Polymers

Two types of reactive acrylic copolymer dispersions were synthesized via seeded emulsion polymerization for use as PA-modified binders. One copolymer contained the epoxy-functional monomer GMA, while the other incorporated the alkoxysilane monomer VTES. Both syntheses followed the same procedure. The ratio of reactive monomer was 5 wt% of all monomer composition. The choice of 5 wt% reactive monomer (GMA or VTES) was guided by our previous optimization studies on epoxy- and silane-functional acrylic copolymers. In earlier research [5,28,29], higher levels of reactive monomers (up to 15 wt%) were successfully employed in core–shell latex systems; however, when incorporated into randomly copolymerized structures, the preliminary studies showed that excessive reactive content (>5 wt%) tended to increase brittleness and reduce film flexibility expecially when phytic acid was used as additive. Conversely, lower ratios (<3 wt%) provided limited enhancement in coating adhesion and durability. Therefore, 5 wt% was identified as the optimal composition that maintains a balance between polymer flexibility, colloidal stability, and mechanical performance.

Initially, deionized water was charged into a four-neck glass reactor and mixed with the anionic surfactant, the nonionic surfactant, and sodium bicarbonate (NaHCO_3_) as buffer. The mixture was homogenized at room temperature for 15 min to form a monomer-free pre-emulsion. The system was then heated to 80 °C in a glycerine bath under mechanical stirring, after which a small portion of APS initiator solution was added to start the reaction. After approximately 5 min, the full monomer mixture (MMA, BA and either GMA or VTES) and the remaining APS solution were simultaneously fed into the reactor from two separate dropping funnels over a period of ~3 h. Upon completion of the feed, the polymerization was allowed to continue for an additional 90 min at 80 °C to ensure complete conversion. The latex was then cooled to room temperature, and the pH was adjusted to 7.0–7.5 with a trietanolamine solution.

The resulting dispersions were stable, bluish-white latexes with a solids content of 25 ± 1 wt%. The two reactive copolymer dispersions—designated G0 (GMA-containing) and V0 (VTES-containing)—were subsequently blended with various amounts of aqueous PA solution and evaluated as binders in leather finishing applications. The polymerization recipe is provided in Table 1.

### 2.3. Leather Finishing Application

The synthesized reactive acrylic polymer dispersions were evaluated as binders in leather finishing to assess their film-forming and coating performance. To clearly observe the influence of the binders, the finishing formulation was kept as simple as possible (Table 2). PA was incorporated into the reactive polymer dispersions at different levels: for the GMA-containing polymer (G0), PA was added at 5, 10, and 15 wt% based on polymer solids, while for the VTES-containing polymer (V0), additions of 5 and 10 wt% were prepared (Table 3). Incorporation levels above 10 wt% in V0 resulted in stability issues.

Finishing trials were performed on brown crust leathers (shoe upper grade, A4 size). The finishing mixtures were applied to the leather surface using a manual spray gun. After each spray coat, the leathers were dried under controlled temperature and time conditions, followed by hot pressing at defined stages to ensure uniform adhesion of the finish film to the substrate. After finishing, the coated leathers were conditioned for several days under standard atmospheric conditions, cured at 100 °C for 30 min, and then reconditioned at room temperature for 24 h prior to physical and surface performance testing. The curing temperature was selected to be adequate for initiating post-crosslinking reactions involving epoxy and silanol functionalities. At this temperature, phytic acid could catalyze epoxy ring-opening and promote silanol condensation, forming covalent or Si–O–P hybrid linkages without causing thermal degradation of the leather substrate.

### 2.4. Characterization Methods

The particle size distribution of the synthesized polymer dispersions and zeta potential values of the latex-phytic acid blends were evaluated with a NanoZS Zetasizer (Malvern Instruments, Worcestershire, UK) after diluting the latex samples 1:500 (*v*/*v*) with distilled water.

The chemical composition of the resulting polymer films was characterized by attenuated total reflectance Fourier-transform infrared (ATR-FTIR) spectroscopy using a Bruker spectrometer (Bruker, Billerica, MA, USA) over a wavenumber range of 450–4000 cm^−1^.

Thermal transitions were analyzed by differential scanning calorimetry (DSC) on a TA Instruments Q2000 unit, applying a heating rate of 10 °C min^−1^ under nitrogen between –60 and 200 °C.

To evaluate the effect of phytic acid incorporation on the water absorption and solvent swelling behavior of the coatings, the reactive binders were blended with phytic acid at the same ratios given in Table 3. Films of each formulation were cast in Petri dishes, dried at room temperature, and subsequently cured at 100 °C for 30 min. After curing, the films were cooled to room temperature in a desiccator, cut into 2 × 2 cm specimens, and accurately weighed (*w_0_*).

For the water uptake test, the samples were immersed in distilled water at room temperature and periodically removed after 30 min, 1 h, 3 h, 6 h, 12 h, and 24 h. The surface water was gently blotted with filter paper, and the wet mass (*wₜ*) was recorded. The water uptake percentage was calculated using the equation:

(1) $$\mathrm{Water}\;\mathrm{Uptake}\;(\%)\;=\;\frac{w_{t}-w_{0}}{w_{0}}\;\times \;100$$

Similarly, acetone swelling tests were conducted under the same conditions to assess the network density and solvent resistance of the films. After immersion for the designated time intervals, the films were wiped, weighed, and the swelling degree was determined analogously. All measurements were performed in triplicate, and average values were reported.

Coating performance was evaluated using standard leather testing protocols, including flexing endurance [30], color fastness to rubbing [31], water spot resistance [32], and heat fastness [33]. Color changes were graded against the Grey Scale Standards [34,35], with scores from 1 (severe change) to 5 (no visible change).

## 3. Results and Discussions

### 3.1. Particle Size and Zeta Potential of the Latexes

Latex stability is a key factor in emulsion polymerization, particularly when reactive monomers such as GMA and VTES are involved. The epoxy rings (oxirane) of GMA and the alkoxysilane groups of VTES are highly susceptible to side reactions such as hydrolysis, condensation, or nucleophilic ring-opening, which can trigger premature crosslinking, gel formation, and coagulation during synthesis. To suppress these undesired reactions and ensure the formation of stable polymer dispersions, the polymerization was carefully controlled. All reactions were carried out in a buffered medium to maintain a slightly alkaline medium, which stabilizes the ionic environment and reduces the risk of uncontrolled hydrolysis. A slow monomer feed strategy and moderate reaction temperatures were employed to further minimize localized concentration gradients and thermal hotspots that could promote gelation. Under these optimized conditions, the final solid content of both latexes was maintained at approximately 25%, allowing efficient polymerization without compromising colloidal stability.

Using this approach, two types of reactive acrylic copolymer dispersions were successfully synthesized: G0, containing GMA as the epoxy-functional monomer, and V0, incorporating VTES as the silane-functional monomer. Both dispersions were obtained as stable, coagulum-free latexes with high polymerization yields exceeding 98%, demonstrating the effectiveness of the controlled process in preventing premature crosslinking.

**Figure 1:**
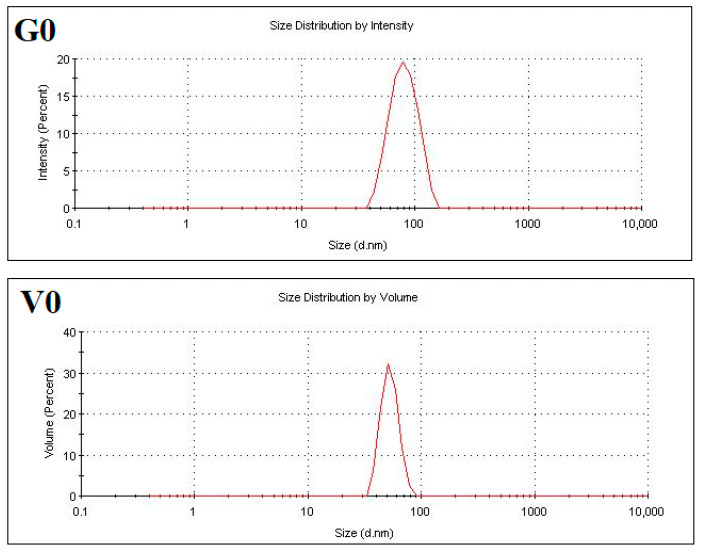
Particle size distribution curves of the latexes.

The particle size distributions of the dispersions were characterized by dynamic light scattering (DLS), and the results are summarized in Figure 1 and Table 4. The G0 dispersion exhibited a Z-average particle diameter of 82 nm with a polydispersity index (PDI) of 0.145, while the V0 dispersion showed a smaller average particle size of 53 nm with a PDI of 0.088. These data indicate that both systems consist of fine particles with narrow and relatively uniform size distributions, confirming the successful preparation of stable, nanoscale reactive latexes suitable for subsequent PA incorporation and leather finishing applications.

The markedly smaller mean particle size measured for the V0 (VTES-containing) dispersion (53 nm, PDI 0.088) compared with G0 (GMA-containing, 82 nm, PDI 0.145) can be ascribed to differences in monomer chemistry that govern nucleation and interfacial behavior during seeded emulsion polymerization. The higher hydrophobicity and lower aqueous solubility of VTES favor the generation of a greater number of primary nuclei, producing a larger particle population with smaller diameters. Moreover, the possible partial hydrolysis of alkoxysilane groups during polymerization generates silanol moieties that increase hydrophilicity and facilitate hydrogen bonding with water molecules. This phenomenon enhances colloidal stabilization by reducing interfacial tension and promoting nucleation over particle growth, ultimately yielding smaller and more narrowly distributed latex particles. Similar effects of silane-induced stabilization have been reported for vinyl silane and organosilane-modified acrylic systems [36,37,38].

Zeta potential analysis was performed to further assess the electrostatic stability and effect of phytic acid addition on the colloidal stability of the reactive acrylic dispersions. The measured zeta potential values are presented in Table 5. For the GMA-containing series, the zeta potential decreased progressively from –51.0 mV (G0) to –27.4 mV (G3) with increasing phytic acid concentration. A similar decreasing trend was observed for the VTES-based series, where the potential dropped from –65.0 mV (V0) to –35.4 mV (V2).

The reduction in surface charge magnitude indicates partial neutralization of the negatively charged latex particles by the acidic phosphate groups of phytic acid. This charge screening effect reduces the electrostatic repulsion among particles, thereby decreasing colloidal stability at higher PA loadings. The results are consistent with the observed sedimentation tendency in formulations containing ≥10 wt% PA, particularly in the VTES system, where silanol condensation and reduced surface charge may jointly contribute to instability.

Overall, the zeta potential data confirm that the latexes exhibit sufficient electrostatic stabilization (|ζ| > 30 mV) up to 10 wt% phytic acid addition, beyond which the dispersion stability begins to decline significantly.

### 3.2. Structural Analyses of Reactive Copolymers and Crosslinked Films

**Figure 2:**
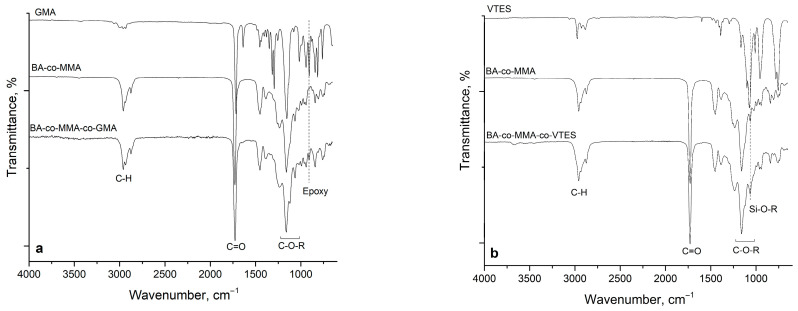
The FTIR spectra of the copolymers and their corresponding reactive monomers (**a**) G0, (**b**) V0.

The FTIR spectra of the copolymers and their corresponding reactive monomers are presented in Figure 2. To verify the incorporation of the reactive monomers into the copolymer structure, a reference BA-co-MMA copolymer was synthesized under identical conditions without any reactive monomer. Its spectrum was recorded for comparison alongside those of the functional monomers.

The spectra exhibit the characteristic absorption bands of poly(acrylate) structures, including the asymmetric and symmetric C–H stretching vibrations of CH_2_ and CH_3_ groups at 2959, 2934, and 2875 cm^−1^; the ester carbonyl (C=O) stretching at 1728 cm^−1^; and the CH_2_/CH_3_ deformation bands at 1452 and 1385 cm^−1^ [39]. The C–O–C stretching vibrations of the ester moiety appear at 1160 and 1066 cm^−1^, confirming the successful formation of the poly(acrylate) backbone [40]. Importantly, no absorption was detected in the 1608–1634 cm^−1^ region corresponding to the vinyl C=C bonds of unreacted acrylate monomers, indicating nearly complete double-bond conversion and effective emulsion polymerization.

Distinct features attributable to the reactive comonomers further validate their incorporation. The characteristic oxirane-ring absorption of GMA is observed at 908 cm^−1^, which also appears in the spectrum of the epoxy-functional copolymer (G0), demonstrating that the epoxide groups remain largely intact and available for subsequent crosslinking [41]. Similarly, the strong Si–O–R stretching band of VTES, centered at ≈1073 cm^−1^, is retained in the V0 copolymer, contributing to an increased intensity of the adjacent C–O stretching band near 1066 cm^−1^ [42]. These spectral signatures confirm the intended copolymer compositions and the preservation of the targeted reactive functionalities in both latex systems.

**Scheme 1:**
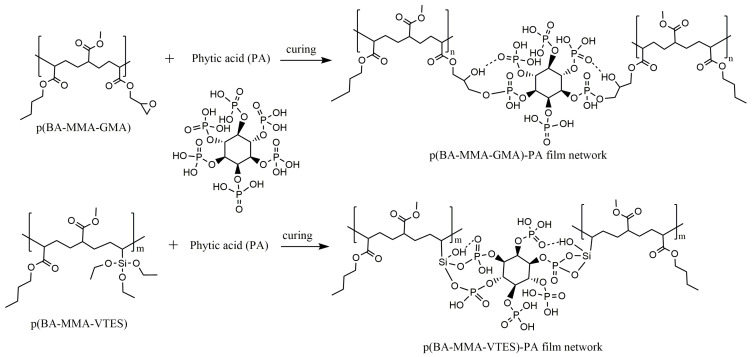
Possible crosslinking mechanism between epoxy and alkoxsysilane functional groups of copolymers.

The multifunctional phosphate groups of phytic acid are capable of forming both covalent and non-covalent interactions with the reactive functionalities present in the acrylic copolymers. Scheme 1 explains the crosslinking mechanism of phytic acid with functional polymer moieties. The phosphate oxygens can act as nucleophiles, attacking the electrophilic carbon of the epoxy ring in GMA units, leading to ring opening and formation of stable P–O–C bonds. Simultaneously, hydrogen bonding between the phosphate and hydroxyl groups can contribute to secondary network reinforcement. In VTES-containing systems, partial hydrolysis of alkoxy groups generates silanol (Si–OH) sites that can undergo condensation with phosphate groups of PA, yielding Si–O–P or Si–O–C linkages. These interactions are expected to enhance crosslinking density and interfacial adhesion, thereby improving the mechanical robustness, rub fastness, and thermal stability of the resulting coating films.

To further evaluate the post-crosslinking behavior of the reactive functionalities, the reactive latexes were mixed with phytic acid with the ratios given at Table 3. The mixtures were then dried at room temperature for 3 days and FTIR analyses were conducted on films containing GMA and VTES, both with and without phytic acid, before and after thermal curing at 100 °C for 30 min.

**Figure 3:**
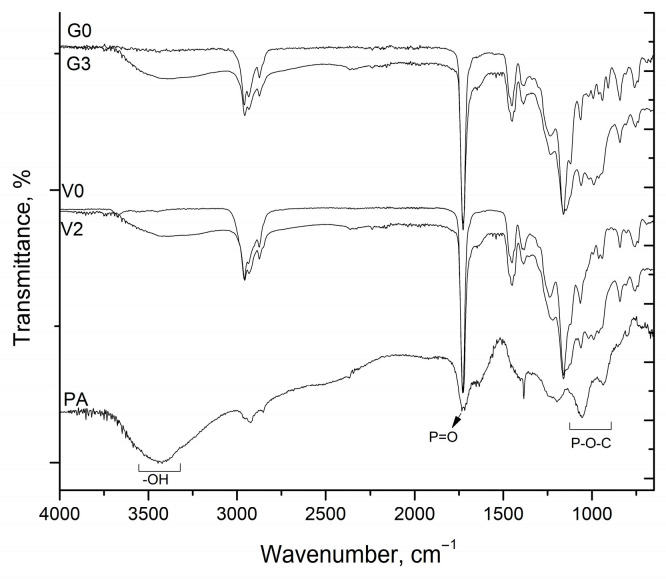
FTIR spectra of phytic acid (PA), pure copolymers (G0,V0), and representative phytic acid incorporated copolymers (G3,V2).

The FTIR spectra of pure phytic acid (PA), unmodified copolymers (G0 and V0), and representative PA-modified copolymers (G3 and V2) are shown in Figure 3. The spectrum of PA exhibited characteristic bands at 3430, 1727, 1193, 1055, and 939 cm^−1^, corresponding to O–H stretching, P=O stretching, P–O–C asym, P-O-C sym stretching and P–O-H stretching vibrations of phosphate groups, respectively. Upon incorporation of PA into the reactive acrylic dispersions, the spectra of G3 and V2 displayed increased band intensities in the regions 3450 cm^−1^ due to the O-H stretching, 1200–900 cm^−1^, particularly around 1055 cm^−1^, which can be attributed to the overlapping of P–O–C and P–O–H vibrations from PA with the C–O stretching of the acrylic backbone. These intensity enhancements confirm the successful incorporation of PA within the polymer matrices.

**Figure 4:**
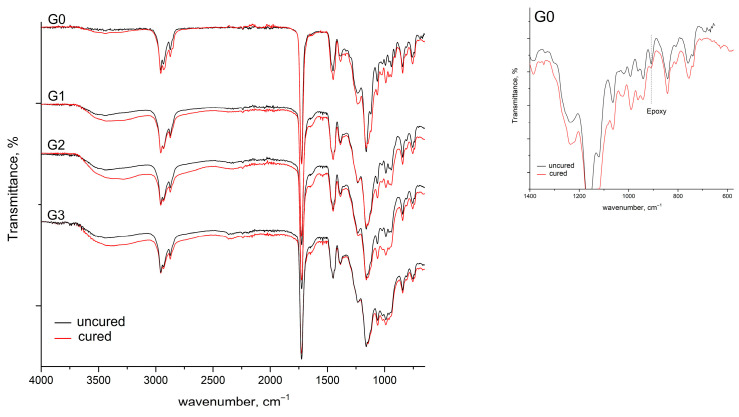
FTIR spectra of PA incorporated GMA-copolymers before and after curing.

Figure 4 represents IR spectra G-series reactive copolymer-PA blends before and after curing process. For the unmodified binder (G0), the FTIR spectrum exhibits a distinct absorption band at 908 cm^−1^, attributed to the epoxy ring stretching of glycidyl methacrylate units. This peak remains visible even after the curing process at 100 °C for 30 min, indicating that thermal treatment alone is not sufficient to induce complete epoxy ring opening in the absence of reactive species. In contrast, the PA-incorporated systems (G1–G3) show a significant reduction or disappearance of the epoxy signal both before and after curing. This observation suggests that phytic acid facilitates partial ring opening reactions, likely through nucleophilic attack of phosphate oxygen atoms on the epoxy groups, leading to the formation of phosphate–ether linkages even at room temperature. Upon curing, a further consolidation of these interactions occurs, as evidenced by increased band intensity in the P–O and C–O stretching region (1050–950 cm^−1^). These spectral changes support the presence of chemical crosslinking between phytic acid and epoxy functionalities, enhancing network formation and contributing to improved film performance.

**Figure 5:**
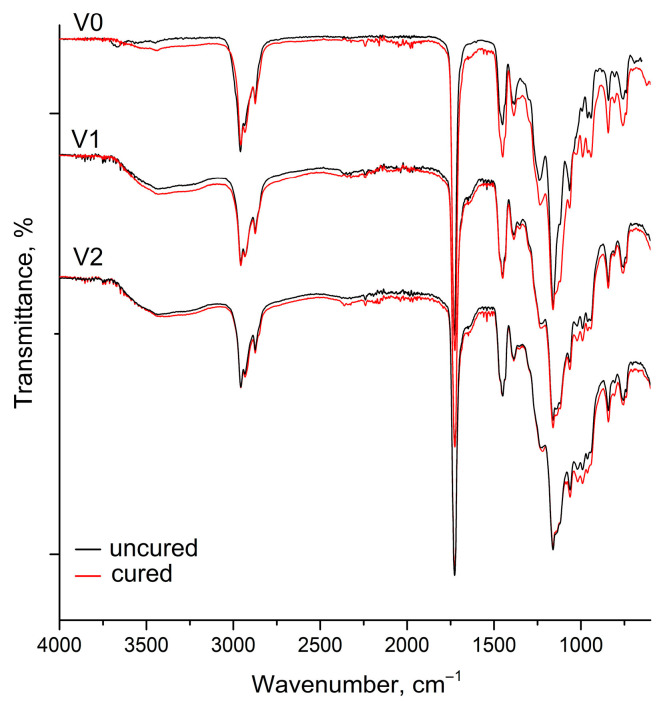
FTIR spectra of PA incorporated VTES-copolymers before and after curing.

The IR spectra of V-series reactive copolymer-PA blends before and after curing process is shown in Figure 5. In the unmodified VTES-containing binder (V0), curing results in a general increase in the absorption intensity within the 1450–750 cm^−1^ region, particularly around 1100 cm^−1^, corresponding to Si–O–Si stretching vibrations. This indicates that alkoxysilane hydrolysis and condensation reactions are activated during the curing process, forming a siloxane network that reinforces the polymer matrix. In PA-modified samples (V1 and V2), this region becomes broader and more intense, suggesting the coexistence of Si–O–Si, Si–O–C, and P–O–Si vibrations. The observed spectral evolution implies that phytic acid participates in silanol–phosphate coordination or condensation reactions, resulting in a denser and more crosslinked hybrid network. Consequently, these chemical interactions are expected to enhance both the structural cohesion and the environmental resistance of the resulting coating films.

### 3.3. DSC Analysis

**Figure 6:**
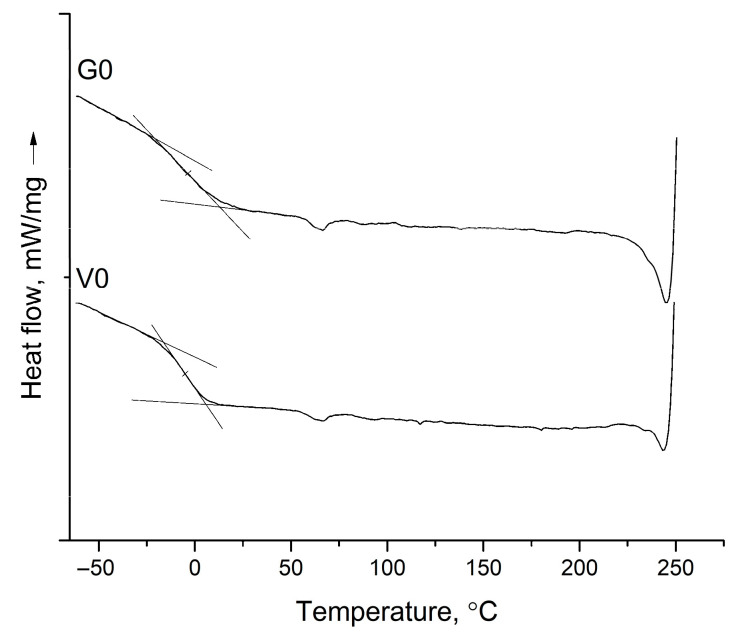
DSC thermograms of copolymers.

The thermal transitions of the neat copolymer films were analyzed by DSC over the –60 to 250 °C range under nitrogen (Figure 6). Both G0 and V0 exhibited a single, broad baseline shift—between −23 and +20 °C for G0 and −22 and +14 °C for V0—attributable to the glass transition of the poly(acrylate) backbone, confirming the amorphous single-phase nature of the copolymers. The midpoint glass-transition temperature (Tg) of G0 (–3.7 °C) was slightly higher than that of V0 (−5.2 °C), consistent with the presence of the more rigid glycidyl methacrylate segments in G0, whereas incorporation of VTES imparts a mildly plasticizing effect. A weak endothermic event near 66 °C was observed for both samples, which is most plausibly associated with residual bound water. At higher temperatures, both copolymers showed a sharp exothermic deviation beginning at approximately 240–250 °C, corresponding to the onset of thermal degradation of the poly(acrylate) backbone. Importantly, no distinct exothermic peak was detected in the 150–200 °C region, indicating negligible post-polymerization crosslinking of either the GMA epoxide or VTES silane functionalities under the applied heating conditions. These results demonstrate that both latexes were obtained as amorphous, single-phase poly(acrylates) without crosslink formation during polymerization, ensuring the availability of reactive groups for subsequent finishing-stage crosslinking.

### 3.4. Water and Solvent Absorption Behavior

The water absorption and acetone swelling behavior of the films are summarized in Table 6 and Table 7. The unmodified copolymer films (G0 and V0) exhibited high water uptake, reaching 35.6 wt% and 41.0 wt%, respectively, after 24 h of immersion. Incorporation of phytic acid (PA) significantly influenced the hydrophilicity and network density of the coatings. At moderate PA loadings (G1–G2 and V1), the overall water absorption decreased, particularly at short immersion times, indicating that PA contributed to the formation of denser polymer networks through covalent/hydrogen bonding or ionic interactions between phosphate groups and reactive functional sites (epoxy or silanol). This denser structure limited the diffusion of water molecules within the film matrix.

At higher PA concentrations, however, the opposite trend was observed. The G3 and V2 samples displayed a marked increase in water uptake (38.1 wt% and 88.3 wt% after 24 h, respectively), suggesting that excessive PA addition introduces unbound hydrophilic phosphate groups and disrupts colloidal uniformity, thereby facilitating water penetration. The strong hydrophilicity of PA and its partial incompatibility at high contents may lead to phase heterogeneity and enhanced water sorption.

Acetone swelling tests further supported these findings. The control samples (G0 and V0) were completely disintegrated in acetone, indicating poor solvent resistance due to the absence of sufficient crosslinking. Upon PA incorporation, all films exhibited a considerable reduction in acetone-induced dissolution, instead showing measurable swelling degrees that gradually reached equilibrium after 6 h. Among the G-series, G1 and G2 displayed moderate swelling ratios (~277–291 wt%), while G3 reached over 320 wt%, consistent with the excessive hydrophilic character at high PA loadings. The V-series showed a similar pattern, with V1 exhibiting the lowest swelling degree (≈239 wt%) and V2 the highest (≈279 wt%).

Overall, these results confirm that moderate incorporation of phytic acid improves film compactness and solvent resistance through controlled interfacial crosslinking, while excessive amounts lead to over-hydrophilization and structural loosening of the polymer network.

### 3.5. Finishing Application Results

#### 3.5.1. Visual Assessment of Finished Leathers

The synthesized reactive acrylic polymer dispersions were applied as finishing coatings onto shoe upper leathers. Each polymer was used individually as the sole binder in simplified finishing formulations to enable a clear assessment of its independent coating performance.

The finishing mixtures were prepared by blending the reactive binders with pigment, wax, isopropyl alcohol (IPA), and aqueous PA solutions at various concentrations. During formulation, it was observed that a high PA content (15 wt% based on polymer solids) caused precipitation in the VTES-containing dispersion; therefore, only 5 and 10 wt% PA levels (samples V2 and V3) were employed for this system. The instability observed in VTES-based dispersions at phytic acid (PA) concentrations above 10 wt% can be attributed to pH-induced hydrolysis and condensation of the silane groups. The introduction of PA lowers the system pH, which accelerates the hydrolysis of the triethoxysilane moieties to silanols. These reactive silanol groups may undergo uncontrolled condensation (Si–O–Si crosslinking) both within and between latex particles, leading to partial aggregation and loss of colloidal stability. Additionally, the high ionic strength introduced by the phosphate groups of PA may disrupt the electrostatic stabilization of the polymer particles, further promoting coagulation. In contrast, the GMA-based dispersions showed no such instability and were formulated at all PA levels. All mixtures were filtered prior to application and sprayed onto the leathers using a hand-held spray gun.

After application, the coatings produced a uniform color distribution with a soft, smooth hand and an attractive medium gloss, indicating good film formation on the leather surface. To ensure completion of any latent reactions, the finished leathers were rested for a short period, heat-treated at 100 °C for 30 min, and then reconditioned under standard testing conditions before being subjected to physical performance evaluations.

#### 3.5.2. Color Fastness to Rubbing

Color fastness to rubbing is a critical parameter for evaluating the performance of leather finishes, as it simulates the potential color transfer that may occur when finished leathers come into contact with other surfaces (e.g., cotton or synthetic fabrics, felt) during use.

In this study, the rub fastness of the coated leathers was assessed according to international standards using both dry and wet rubbing tests. In each test, a white felt pad was rubbed back and forth across the finished leather surface under a defined load and number of cycles. After testing, the felt was examined for color transfer, while the leather surface itself was inspected not only for color change but also for possible physical defects such as gloss loss, surface dulling, or film peeling. All color changes were rated using the ISO Grey Scale under a daylight-simulated light cabinet. The results are summarized in Table 8.

In the dry rubbing test, all samples withstood up to 2000 cycles. The epoxy-containing control sample (G0) received a rating of 3 (leather) and 3/4 (felt) after 2000 dry cycles. The incorporation of PA further enhanced dry rub resistance, and a similar trend was observed for the VTES-based coatings.

**Figure 7:**
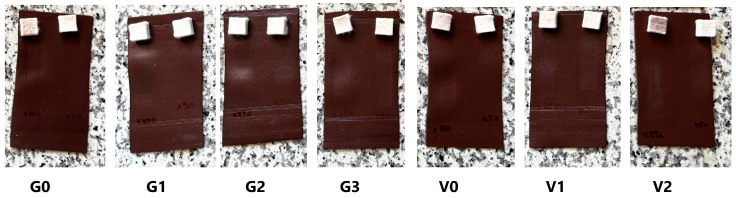
The images of finished leathers after wet rubbing test.

Performance differences became more pronounced in the wet rubbing test (Figure 7). At 50 wet cycles, all samples exhibited good resistance. After 250 wet cycles, the G0 sample showed ratings of 3 (leather) and 2/3 (felt), which increased markedly with the addition of PA up to 10 wt%. However, further increases in PA content led to a gradual decline in wet rub fastness. A comparable behavior was observed for the VTES-based finishes. For example, the fastness level of V0 improved from 2/3 (leather) and 3 (felt) to 4 (leather) and 3/4 (felt) with the addition of 5 wt% PA, but higher PA loadings resulted in reduced ratings, dropping as low as 1/2.

Overall, the incorporation of 5 wt% PA provided the most balanced improvement in rub resistance for both systems. Additionally, epoxy-based coatings consistently exhibited higher rub fastness values than the silane-containing finishes under both dry and wet conditions. It should be noted that the coatings exhibited superior resistance under both dry and wet rubbing conditions—enduring up to 2000 and 250 cycles, respectively, which exceed the standard test cycles typically used for footwear leathers (500 dry, 50 wet). This superior rub fastness is likely the result of a synergistic effect between covalent and secondary interactions. During curing, phosphate groups of PA may react with epoxy and silanol moieties, yielding C–O–P or Si–O–P linkages, while simultaneously forming hydrogen bonds and ionic associations with polar sites naturally present in the collagen matrix and/or with the chromium complex of tanned leather. These combined effects improve interfacial adhesion and film cohesion, leading to enhanced mechanical durability. However, at higher PA levels, the excessive hydrophilicity and potential plasticizing effect of unreacted PA likely compromise network compactness and reduce film cohesion, leading to slightly decreased wet rub fastness.

#### 3.5.3. Flex Resistance of Finished Leathers

The results of the flexometer (bending) test are summarized in Table 9. Flex testing is a key performance evaluation for shoe upper leathers, as it simulates the repeated bending and stretching that occurs during walking and assesses the resistance of the finish layer to cracking or other mechanical damage. Each sample was first subjected to 50,000 flex cycles, followed by an extended test up to 100,000 cycles.

Across all formulations, no peeling, blistering, or visible color change was observed on the finished surfaces even after 100,000 cycles. Minor surface wrinkling was noted in some samples during flexing, and this effect became slightly more pronounced with increasing PA content. The flexometer test provides a standardized assessment of visible defects and failures in the leather finish through qualitative evaluation of surface integrity. However, developing instrumental approaches for quantifying flex damage (e.g., optical or microscopic surface analysis) could be an interesting direction for future research. Nevertheless, all coated leathers met the flex performance requirements, demonstrating that both the epoxy- and silane-based reactive binders provided adequate flexibility and durability for demanding footwear applications.

#### 3.5.4. The Water Spotting Test Results

The water spot test evaluates a leather finish’s ability to withstand moisture-related effects such as staining, surface marking, blistering, or color alteration. To simulate prolonged contact with water, two drops of distilled water were placed on the finished leather surface. After 30 min, excess water from one drop was gently removed with filter paper and the surface was inspected for any visible changes. The second drop was left to evaporate overnight, and color variation was assessed using the standard grey scale after 16 h of exposure.

**Figure 8:**
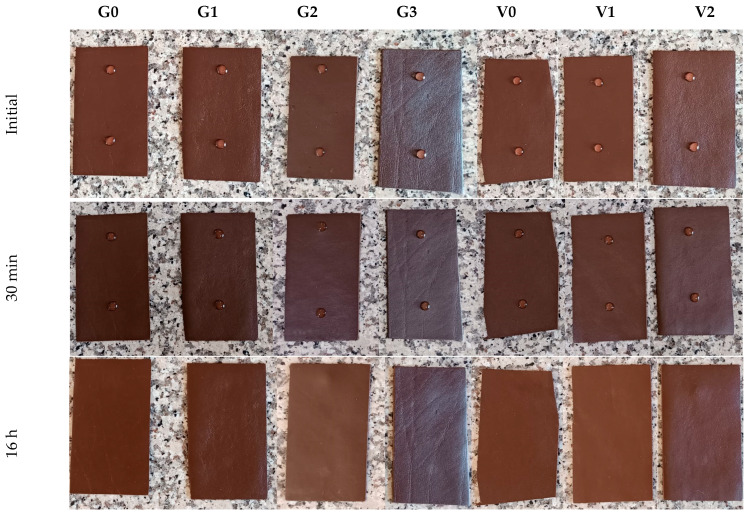
The images of finished leather at different stages of water spotting test.

The results of the water drop (water spot) test after 30 min and 16 h are summarized in Table 10, and representative images taken after 30 min are shown in Figure 8. Even after the first 30 min of exposure, the water droplets remained on the leather surface without being absorbed, and removal of the initial drop caused no visible physical damage or noticeable surface change. This behavior demonstrates the high water repellency of the finish films across all samples.

After 16 h, no significant color change was detected on any of the finished leathers; only a very light watermark was observed on samples containing PA. Overall, the finished leathers exhibited excellent resistance to water spotting, confirming the strong protective barrier provided by the reactive polymer coatings.

#### 3.5.5. Heat Resistance of Finished Leathers

The heat resistance of the finished leathers was evaluated by applying heated chrome plates at 150, 200, 250, and 300 °C under a pressure of 0.21 kg/cm^2^ for 5 s. Following exposure, the samples were examined under a light cabinet using the grey scale to assess color changes and other surface effects. The evaluation results are summarized in Table 11.

As expected, increasing temperature led to gradual color changes on the leather surfaces. For the GMA-based coatings, the incorporation of PA provided a noticeable improvement in thermal stability, particularly in the formulation containing 10 wt% PA (G2), which showed the best resistance at elevated temperatures. In contrast, PA addition did not produce a significant effect on the thermal resistance of the VTES-based coatings.

Overall, all samples exhibited good heat resistance, with no severe film failure or finish delamination observed even at 300 °C. At this highest temperature, only slight shrinkage of the leather substrate was detected, confirming that the coatings retained their structural integrity under extreme thermal stress.

## 4. Conclusions

Two reactive acrylic copolymer dispersions containing epoxy (GMA) and alkoxysilane (VTES) functionalities were successfully synthesized via seeded emulsion polymerization and subsequently modified with phytic acid (PA) at varying concentrations for use as binders in footwear leather finishing. Both dispersions exhibited high polymerization yields (>98%), fine particle size, and good colloidal stability, as verified by DLS and zeta potential measurements. FTIR analyses confirmed the successful incorporation of the reactive groups and provided evidence of interactions between phytic acid and functional monomers, suggesting the occurrence of covalent or coordinative crosslinking.

The water uptake and acetone swelling experiments further supported these findings, indicating that moderate PA incorporation (5–10 wt%) improved film cohesion and solvent resistance by enhancing the crosslinking density, while excessive PA levels led to network heterogeneity and reduced stability. Performance evaluations showed that the reactive binders provided uniform film formation, good surface appearance, and high fastness properties on finished leathers. Rub fastness remained excellent under both dry and wet conditions up to 10 wt% PA addition, although higher PA levels reduced wet rub resistance and dispersion stability, particularly in the VTES system. Flexing endurance tests confirmed strong resistance to repeated bending, with only minor surface wrinkling observed at higher PA contents. Water spot tests indicated very good resistance across all samples, while heat resistance assessments revealed improved thermal stability in the GMA-based binders with increasing PA content.

Overall, the results demonstrate that PA-modified reactive acrylic binders combine strong performance with enhanced environmental compatibility, offering a promising alternative to conventional toxic crosslinkers in leather finishing. The developed systems are waterborne and solvent-free, relying on bio-based, non-toxic phytic acid instead of hazardous aziridine or isocyanate crosslinkers, thereby minimizing VOC emissions and improving workplace and ecological safety. Optimized PA incorporation enables retention of coating performance while contributing to sustainability objectives, underlining the potential of such reactive and bio-derived systems for next-generation, high-performance, and environmentally responsible leather finishing technologies.

## Figures and Tables

**Table 1:** The polymerization recipe.

Components	Amount (g)
BA	13.93
MMA	7.60
GMA/VTES	1.17
Emulsogen EPN 287	1.89
Sulfopon^®^ 101 UP	2.37
NaHCO_3_	0.03
APS	0.13
Water	72.88
Total	100.00

**Table 2:** The finish formulation applied on the leathers.

Components	Application Steps		Descriptions
	Basecoat (I) (Parts)	Topcoat (II) (Parts)	
Water	48	20	Spray I ×3 times
Pigment	10	-	Hot plate 90 °C/100 bar
Wax	5	-	
Reactive Polymer Emulsion(G0/V0)	30	-	Spray I ×2 times
Isopropyl alcohol	0.5	-	Hot plate 90 °C/70 bar
PA	x *		
Aqueous NC Lacquer	-	10	Spray II ×2 times

* amount is varied (Table 3).

**Table 3:** Finishing formulations with varying PA content.

Finishing Mixture	PA Content * (wt%)
G0	0
G1	5
G2	10
G3	15
V0	0
V1	5
V2	10

* Calculated based on the solid weight of the binder. G series: Prepared using the GMA-containing reactive polymer emulsion. V series: Prepared using the VTES-containing reactive polymer emulsion.

**Table 4:** The average particle sizes and size distributions of the latexes.

Polymer Sample	Average Particle Diameter (nm)	Polydispersity Index (PDI)
G0	82.1 ± 5.3	0.145 ± 0.020
V0	53.3 ± 6.6	0.088 ± 0.034

**Table 5:** The zeta potential values of the latexes containing different ratios of PA.

Latex/PA Blends	Zeta Potential Value (ζ, mV)
G0	−51.0 ± 2.3
G1	−43.5 ± 1.1
G2	−37.2 ± 0.9
G3	−27.4 ± 1.5
V0	−65.0 ± 1.2
V1	−55.1 ± 1.0
V2	−35.4 ± 1.6

**Table 6:** Water absorption behavior of phytic-acid-modified and unmodified reactive acrylic copolymer films at different immersion times in distilled water (room temperature).

Samples	Water Uptake (wt%)					
	0.5 h	1 h	3 h	6 h	12 h	24 h
G0	5.7	13.3	14.9	20.1	26.8	35.6
G1	4.5	7.6	17.3	22.0	23.1	25.4
G2	7.8	8.8	12.5	17.2	18.0	20.5
G3	11.2	15.0	26.6	36.5	38.2	38.1
V0	6.3	8.6	12.6	21.6	31.7	41.0
V1	2.2	3.0	11.7	18.6	26.5	32.8
V2	9.4	14.3	38.8	57.1	73.3	88.3

**Table 7:** Solvent swelling behavior of phytic-acid-modified and unmodified reactive acrylic copolymer films during immersion in acetone at room temperature.

Samples	Acetone Swelling (wt%)					
	0.5 h	1 h	3 h	6 h	12 h	24 h
G0	-	-	-	-	-	
G1	220.9	262.8	275.8	277.0	277.4	277.7
G2	283.3	287.0	287.8	288.4	289.5	290.9
G3	293.0	295.7	314.8	315.9	318.9	321.4
V0	-	-	-	-	-	-
V1	223.8	225.3	230.4	234.3	237.1	238.8
V2	228.1	239.4	252.9	264.3	271.5	279.3

**Table 8:** Rubbing fastness properties of leather samples coated with hybrid latexes.

Samples	Evaluated Parts	Grey Scale Values			
		Dry		Wet	
		500 Cycles	2000 Cycles	50 Cycles	250 Cycles
G0	Leather	4	3	4	3
	Felt	4	3/4	5	2/3
G1	Leather	5	5	5	4/5
	Felt	5	5	5	5
G2	Leather	5	5	5	4/5
	Felt	5	5	5	4/5
G3	Leather	5	4/5	4	3/4
	Felt	5	5	5	3
V0	Leather	4	3/4	4	2/3
	Felt	4/5	3	4	3
V1	Leather	5	5	4/5	4
	Felt	5	5	5	3/4
V2	Leather	5	4/5	4	1/2
	Felt	5	5	4	1/2

**Table 9:** The flexing endurance results of finished leathers.

Samples	Flexometer	
	×50,000	×100,000
G0	Very light wrinkles	Light wrinkles
G1	light wrinkles	Deep wrinkles
G2	light wrinkles	Deep wrinkles
G3	light wrinkles	Deep wrinkles
V0	light wrinkles	Deep wrinkles
V1	light wrinkles	Deep wrinkles
V2	light wrinkles	Deep wrinkles

**Table 10:** Color fastness of leather samples to water spotting after 30 min and 16 h.

	30 Min		16 h	
Leathers	Observation	Grey Scale Value	Observation	Grey Scale Value
G0	The water drop remained without absorption	5	No spot	5
G1	The water drop remained without absorption	5	Very light spot	4/5
G2	The water drop remained without absorption	4/5	Very light spot	4
G3	The water drop remained without absorption	4/5	Very light spot	4
V0	The water drop remained without absorption	5	No spot	5
V1	The water drop remained without absorption	5	No spot	4/5
V2	The water drop remained without absorption	4/5	Very light spot	4

**Table 11:** Evaluation results of the heat resistance test at different temperatures.

Samples	Gray Scale Ratings			
	150 °C	200 °C	250 °C	300 °C
G0	5	4/5	4	3
G1	5	4/5	4	3/4
G2	5	5	4/5	4
G3	5	4/5	4	3/4
V0	5	5	4/5	4
V1	5	4/5	4	3
V2	5	5	4/5	3

## Data Availability

The data that support the findings of this study are available on request from the corresponding author.
